# Opportunities and Challenges of Implementing Solar-Powered Digital Health in Low-Resource Settings: A Mixed-Methods Study

**DOI:** 10.1177/00469580261485823

**Published:** 2026-09-01

**Authors:** Ronald Danny Nyatuka, Paul Macharia, Md Shafiqur Rahman Jabin

**Affiliations:** 1 School of Computing and Engineering Sciences, 118991 Strathmore University, Nairobi, Kenya; 2 Department of Medicine and Optometry, 4180 Linnaeus University, Kalmar, Sweden

**Keywords:** digital health, solar energy, low-resource settings, health system strengthening, electronic medical records, digital health infrastructure, health information systems, healthcare facilities, mixed-methods research, renewable energy

## Abstract

**Background:**

Digital health systems can improve healthcare delivery in low-resource settings but are often constrained by unreliable electricity and inadequate infrastructure. Solar-powered energy systems offer a promising solution to improve the resilience and sustainability of digital health services. This study evaluated the opportunities, challenges, technical feasibility, economic viability, and health system readiness for implementing solar-powered digital health systems in low-resource settings.

**Methods:**

A cross-sectional mixed-methods study was conducted across 12 public health facilities in rural and peri-urban areas. Quantitative data were collected through structured facility assessments and surveys, while qualitative data were obtained through key informant interviews and observational assessments. Descriptive statistics were used for quantitative analysis, and thematic analysis was applied to qualitative data. Findings were integrated to generate system-level insights.

**Results:**

Although 86% of facilities were connected to the national grid, only 29% reported reliable electricity, and 83% experienced frequent power outages. Limited computing resources (29%) and unstable internet connectivity (60%) further constrained digital health operations. Most facilities (75%) reported high energy-related costs, often due to generator use. Despite these challenges, 92% of respondents supported solar-powered solutions, citing improved service continuity, reduced operational disruptions, and enhanced system performance. Qualitative findings highlighted reliance on paper-based workarounds and emphasized the interdependence of energy, connectivity, and digital infrastructure.

**Conclusion:**

Solar-powered digital health systems represent a feasible and context-appropriate approach for strengthening digital health infrastructure in low-resource settings. However, successful implementation requires coordinated investments addressing technical, financial, and organizational barriers to support long-term sustainability and health system resilience.

## Introduction

### Background

Digital health technologies are increasingly recognized as essential components of health system strengthening, particularly in low- and middle-income countries (LMICs), where persistent challenges in access, quality, and efficiency of care remain. Interventions such as electronic medical records (EMRs), telemedicine, and mobile health (mHealth) applications have demonstrated the potential to improve clinical decision-making, enhance data continuity, and support service delivery across diverse settings.^1-3^ Global policy frameworks further emphasize the role of digital health in advancing universal health coverage and improving health outcomes through integrated, patient-centered systems.^4,5^

Despite these advancements, the implementation and sustainability of digital health systems remain uneven, particularly in resource-constrained settings. Evidence suggests that digital health outcomes are strongly influenced by contextual factors, including infrastructure, governance, and system design, rather than technology alone.^6,7^ This highlights the need for approaches that consider digital health as part of a broader socio-technical system embedded within health service delivery environments.^8-10^

### Infrastructure Constraints in LMICs

Reliable electricity and connectivity are foundational requirements for digital health systems, yet many healthcare facilities in sub-Saharan Africa operate with limited or unstable access to these resources. A systematic review has shown that a significant proportion of health facilities lack consistent electricity, directly affecting the use of medical devices and digital platforms.^11^ Similarly, global energy reports highlight persistent disparities in access to reliable power across LMICs, particularly in rural and underserved areas.^12-15^

These infrastructural limitations directly affect system functionality, including reduced uptime, data loss, and disruptions to clinical workflows. As a result, many facilities continue to rely on hybrid systems that combine digital and paper-based processes, limiting the effectiveness of digital health investments.^6^ Furthermore, connectivity challenges, including low bandwidth and intermittent internet access, constrain the ability to fully utilize digital platforms, particularly those dependent on cloud-based services.^15,16^

### Context of Digital Health Implementation in Kenya and Ethiopia

Kenya and Ethiopia were included as illustrative East African low-resource settings representing diverse infrastructural, operational, and health system contexts in which solar-powered digital health implementation can be examined. The study was not designed as a formal cross-country comparative analysis but rather to generate transferable insights across diverse resource-constrained settings. Kenya has made notable progress in digital health adoption, including implementing electronic medical records (EMRs) and national health information systems in public healthcare facilities.^17-19^ However, disparities persist between urban and rural areas, where infrastructural constraints and contextual barriers continue to affect system performance and integration.^20-22^

Similarly, Ethiopia has made significant strides in expanding primary healthcare and advancing universal health coverage, while continuing to face challenges related to energy access, digital infrastructure, and system interoperability.^23,24^ Together, these settings provide valuable insights into the opportunities and challenges associated with implementing and sustaining digital health interventions in low-resource environments.

### Solar-Powered Digital Health as a Potential Solution

Renewable energy technologies are increasingly being explored as sustainable approaches for supporting healthcare infrastructure and digital health systems in resource-constrained settings. Integrating renewable energy solutions, particularly solar-powered systems, has emerged as a promising approach to address infrastructural barriers in LMICs.^25,26^ Reliable electricity is essential for maintaining digital health systems, and its absence has been identified as a critical barrier to delivering quality healthcare services.^3,27,28^ Solar-powered solutions provide decentralized, sustainable energy sources that support continuous system operation, particularly in off-grid and underserved settings.

When combined with adaptable digital architectures, including offline-capable systems and hybrid connectivity models, solar-powered digital health solutions can enhance system resilience and reduce dependence on unstable infrastructure.^16^ Such approaches align with broader efforts to develop context-sensitive and sustainable digital health interventions in resource-constrained environments.

### Rationale for a Multi-Country Assessment

Despite growing interest in digital health and renewable energy integration, existing evidence largely derives from single-country studies, limiting the generalizability of findings across contexts. Digital health interventions are inherently complex and context-dependent, influenced by variations in infrastructure, workforce capacity, governance structures, and financing mechanisms.^29-31^

Including healthcare facilities from Kenya and Ethiopia enabled the study to capture experiences across diverse East African low-resource settings with differing infrastructural and operational characteristics. Rather than undertaking a formal comparison between countries, the study sought to identify common implementation challenges and opportunities that may inform digital health implementation in comparable resource-constrained contexts. By identifying common challenges and implementation considerations across diverse low-resource settings, this study seeks to generate insights that can inform the sustainable scaling of digital health interventions in LMICs.

### Study Objective

This study aims to assess the opportunities, challenges, and feasibility of implementing solar-powered digital health systems in low-resource settings across Kenya and Ethiopia. Using a mixed-methods approach, the study examines three key domains, i.e., technical feasibility, operational readiness, and economic viability, to identify common opportunities, implementation challenges, and health system considerations across diverse low-resource healthcare settings. The findings are intended to inform context-sensitive strategies for the design, implementation, and scaling of integrated energy, i.e., digital health solutions in resource-constrained settings.

## Methods

The study is reported in accordance with the Good Reporting of A Mixed Methods Study (GRAMMS) recommendations,^32^ and the completed GRAMMS checklist is provided as Multimedia Appendix 1.

### Study Design

This study employed a mixed-methods design to assess the opportunities, challenges, and feasibility of implementing solar-powered digital health systems in Kenya and Ethiopia. Mixed-methods approaches are particularly suitable for evaluating complex health system interventions, as they enable the integration of quantitative and qualitative data to provide a comprehensive understanding of system performance and contextual influences.^33-37^

The mixed-methods design enabled a systematic assessment of structural, infrastructural, and operational factors influencing feasibility across diverse healthcare settings. This approach aligns with implementation research and health systems research methodologies, which emphasize context-sensitive evaluation of interventions in real-world settings.^38-41^

### Study Setting

The study was conducted across selected healthcare facilities in Kenya and Ethiopia, representing diverse rural and peri-urban contexts. These settings were chosen due to their varying levels of infrastructure development, digital health adoption, and health system capacity. Participation was voluntary, and all participants provided informed verbal consent before interviews, focus group discussions, and observational assessments began. Verbal consent was considered appropriate because the study posed minimal risk, involved no clinical intervention or collection of sensitive personal health information, and was conducted in accordance with the ethical approval requirements governing the study. This study was conducted and reported in accordance with relevant guidance for mixed-methods research reporting.

Kenya has demonstrated relatively advanced implementation of digital health systems, including EMRs and national health information platforms.^17^ In contrast, Ethiopia has more constrained infrastructure, particularly in energy access and digital system integration, while continuing to expand primary healthcare services.^23^ Including facilities from both countries broadened the diversity of healthcare contexts represented in the study and enhanced the transferability of the findings to comparable low-resource settings. The study was not designed to perform country-level comparative analyses.^3,15,16,42^

Facilities were purposively selected to represent diverse rural and peri-urban healthcare contexts with varying levels of digital health adoption, infrastructure availability, and health system capacity. Eligible facilities included public health facilities with existing or planned digital health activities and a willingness to participate in the study. Facilities that were inaccessible during the study period or unable to support data collection activities were excluded.

The inclusion of 12 healthcare facilities and seven key informants was considered appropriate for the exploratory mixed-methods design. Facilities were purposively selected to maximize variation in geographical location, level of care, digital health implementation, and infrastructure characteristics, thereby capturing diverse implementation contexts.^37^ The final sample size was also informed by pragmatic considerations, including accessibility, available resources, and the feasibility of conducting in-depth field assessments across multiple sites. For the qualitative component, data collection continued until sufficient information power and thematic saturation were achieved, with no substantially new themes emerging during the later interviews.^43^ This approach is consistent with established guidance for qualitative and mixed-methods health systems research.^34,37^

### Conceptual and Analytical Framework

The study was guided by a systems-oriented and socio-technical perspective, recognizing that digital health systems operate within complex interactions between technology, infrastructure, and organizational processes.^8,9,44,45^

A multi-dimensional feasibility framework was applied, comprising three interrelated domains:

1. Technical feasibility (infrastructure, system performance, connectivity)
2. Operational feasibility (workflow integration, user capacity, institutional readiness)
3. Economic feasibility (costs, sustainability, and financing models)

This integrated approach is consistent with established frameworks for evaluating digital health and complex interventions, which emphasize the importance of considering interdependencies across system components.^30,46-49^

### Data Collection

Data collection was conducted between November 2025 and March 2026 following receipt of ethical approval. Data were collected using multiple complementary methods to ensure triangulation and depth of analysis.

#### Key Informant Interviews

Semi-structured interviews were conducted with healthcare workers, facility managers, policymakers, and technical stakeholders. The interviews explored perceptions of technical feasibility, economic sustainability, and health system readiness. Semi-structured approaches allow flexibility in exploring participant experiences while maintaining consistency across interviews.^50,51^

#### Focus Group Discussions and Stakeholder Workshops

These were used to capture collective perspectives on governance, financing, and system integration. These methods facilitated interaction among participants and enabled the identification of shared challenges and priorities.^52^

#### Facility Observations

Structured and semi-structured observational assessments were conducted to evaluate infrastructure conditions, including power supply, connectivity, and availability of digital systems. Observational methods provided objective insights into real-world system functionality and environmental constraints.^53^

#### Document and Secondary Data Review

Relevant project documents, policy materials, and system performance indicators were reviewed to complement primary data sources. This included assessments of system uptime, data continuity, and infrastructure reliability.

Inclusion criteria comprised public healthcare facilities located in rural and peri-urban settings and participants involved in digital health implementation, facility management, ICT support, or clinical service delivery. Exclusion criteria included facilities that were inaccessible during the study period and individuals not directly involved in digital health, infrastructure management, or service delivery processes.

### Data Analysis

#### Qualitative Analysis

Qualitative data from interviews, focus groups, and open-ended observations were analyzed using thematic analysis, following established approaches for identifying patterns and themes across datasets.^54,55^ The analysis involved familiarization with the data, coding, theme development, and iterative refinement. This approach enabled the identification of key themes related to infrastructure constraints, operational challenges, and contextual factors influencing feasibility.

Semi-structured interview and focus group discussion guides were developed to explore technical feasibility, economic viability, and health system readiness. Facility observations used a structured checklist to assess power supply, ICT infrastructure, digital health systems, workforce capacity, financing arrangements, and organizational readiness.

#### Quantitative Analysis

Quantitative data from facility assessments and system indicators were analyzed descriptively to evaluate infrastructure availability, system performance, and resource distribution across participating facilities. These indicators informed the overall assessment of the feasibility of solar-powered digital health in low-resource settings.

#### Integration of Mixed Methods

Findings from qualitative and quantitative analyses were integrated through triangulation to provide a comprehensive feasibility assessment. Integration of multiple data sources enhances the validity and robustness of findings in mixed-methods research.^35,56^ Quantitative and qualitative findings were integrated during interpretation through triangulation to identify convergent findings, complementary insights, and contextual factors that influence the feasibility of solar-powered digital health systems.

#### Integrated Mixed-Methods Analysis

**Figure 1.**
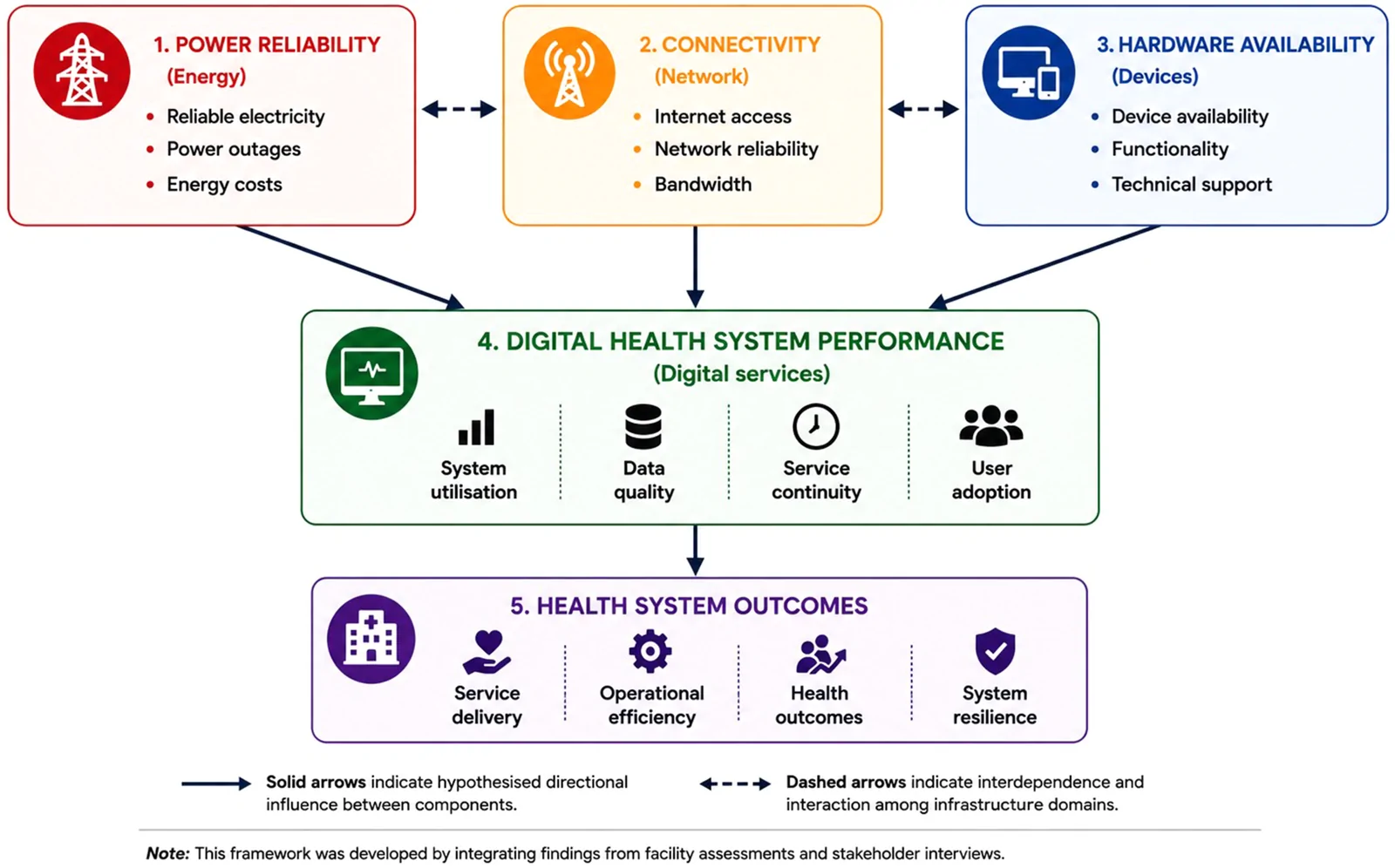
Conceptual framework illustrating the determinants of digital health system performance in low-resource settings

Quantitative and qualitative findings were integrated to develop a comprehensive understanding of the feasibility of solar-powered digital health systems across participating facilities. The analysis examined infrastructure availability, workforce capacity, governance arrangements, financing considerations, and operational challenges to identify factors influencing implementation feasibility. This integrated approach enabled the generation of system-level insights and aligns with established mixed-methods research practices.^57^ A conceptual framework was developed by integrating the quantitative and qualitative findings to illustrate the relationships among key infrastructural determinants of digital health system performance in low-resource settings (see Figure 1).

Figure 1 presents a conceptual framework derived from the integrated quantitative and qualitative findings. The framework illustrates how three interdependent infrastructure domains, i.e., power reliability, connectivity, and hardware availability, influence digital health system performance and, ultimately, health system outcomes. It emphasizes that these domains function as an interconnected system, in which limitations in one domain may adversely affect service continuity, data availability, and overall system resilience. The framework provides a conceptual basis for understanding the infrastructural conditions necessary to support sustainable digital health implementation in low-resource settings.

## Results

### Overview of Study Sample

The study included 12 public health facilities across rural and peri-urban settings, along with 7 key informants who participated in the mixed-methods assessment. The respondent profile was predominantly composed of ICT support personnel (4/7, 57%), with additional representation from clinical officers, nurses, and health records officers (1/7, 14% each). Participants had varying levels of professional experience: 3 of 7 respondents (43%) reported 3–5 years, 2 of 7 (29%) reported 0–2 years, and 2 of 7 (29%) reported more than 5 years.

The included facilities ranged from Level 2 dispensaries to Level 4 hospitals, enabling an examination of feasibility across diverse service delivery and infrastructure contexts. Table 1 summarizes the characteristics of the participating health facilities and their existing digital health infrastructure.

**Table 1.** Facility Characteristics and Digital Health Infrastructure Across Participating Facilities (N=12)

Characteristic	Category	n (%)
Total facilities	Participating facilities	12 (100)
Geographic setting	Rural and peri-urban facilities	12 (100)
Total respondents	Participants	7 (100)
ICT support staff	Respondents	4 (57)
Clinical officers	Respondents	1 (14)
Nurses	Respondents	1 (14)
Health records officers	Respondents	1 (14)
0–2 years of experience	Respondents	2 (29)
3–5 years of experience	Respondents	3 (43)
>5 years of experience	Respondents	2 (29)

### Technical Feasibility

#### Power Reliability and Energy Resilience

Unless otherwise specified, infrastructure, economic, and system-related indicators are reported at the facility level and are based on the 12 participating healthcare facilities (N=12). Quantitative findings indicate that while access to electricity was widespread, reliability was a major constraint. Most facilities (86%) relied on the national grid as their primary power source; however, only 29% reported high reliability, while the remaining 71% reported moderate reliability. Furthermore, 40% of facilities lacked backup power, while 60% relied on generators, often constrained by fuel costs and maintenance challenges.

At a more granular level, 83% of facilities experienced frequent power outages, and over half (58%) reported daily or near-daily outages. These disruptions had direct operational consequences: 67% of facilities reported interruptions in electronic medical record systems during outages, while 50% experienced disruptions to laboratory services and cold chain systems. Table 2 presents key quantitative indicators of electricity reliability, connectivity, hardware availability, and energy-related challenges across participating facilities.

**Table 2.** Key Infrastructure, Economic, and Perception Indicators Across Participating Facilities (N=12)

Domain	Indicator	n (%)
Power	Facilities relying on grid electricity	10 (83)
	Facilities reporting high reliability	3 (25)
	Facilities reporting moderate reliability	9 (75)
	Facilities with no backup power	5 (42)
	Facilities with generators	7 (58)
	Facilities with frequent outages	10 (83)
	Facilities with daily/near-daily outages	7 (58)
	Facilities with EMR disruptions	8 (67)
	Facilities with laboratory or cold-chain disruptions	6 (50)
ICT Infrastructure	Adequate computer access	3 (25)
	Limited computer access	9 (75)
	Stable internet connectivity	5 (42)
	Unstable internet connectivity	7 (58)
Economic	Facilities with unpredictable energy costs	9 (75)
	Facilities relying on generators (cost burden)	7 (58)
	Facilities with unplanned maintenance costs	8 (67)
	Facilities with inconsistent ICT funding	6 (50)
Perception	Perceived benefit of solar energy	11 (92)

NB: Facility-level indicators are based on 12 participating healthcare facilities (N=12). Respondent characteristics are based on 7 key informants (N=7).

Qualitative findings provided critical contextual depth to these patterns. Participants consistently described the power supply as unpredictable and insufficient to sustain routine clinical and digital operations. In several instances, prolonged outages required suspending digital workflows or reverting to paper-based systems. Backup generators were widely perceived as unsustainable due to high fuel costs, while existing solar installations, where present, were typically limited to essential equipment and insufficient to support broader digital infrastructure.

Across both data strands, there was strong convergence regarding the perceived value of solar energy. The overwhelming majority of respondents (92%) indicated that reliable solar-powered systems would significantly enhance service continuity, stabilize digital platforms, and reduce operational disruptions. These findings position energy reliability not merely as an infrastructural issue but as a foundational determinant of digital health system performance.

**Figure 2.**
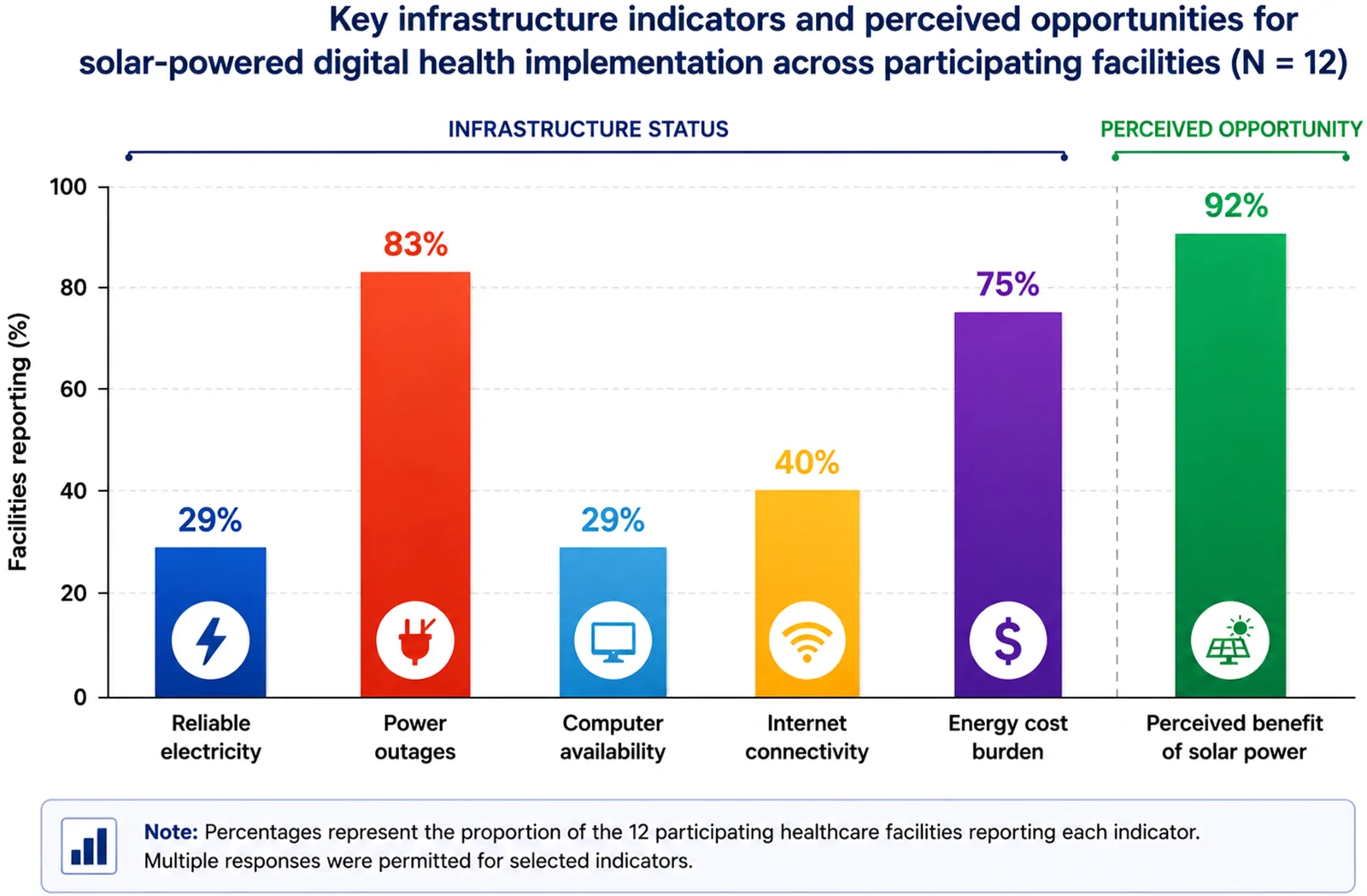
Key infrastructure indicators and perceived opportunities for solar-powered digital health implementation across participating healthcare facilities (N = 12)

Figure 2 summarizes the key quantitative indicators related to infrastructure availability, system constraints, and perceived opportunities for solar-powered digital health systems across participating facilities.

Figure 2 presents the key quantitative indicators of digital health infrastructure across participating healthcare facilities. The findings indicate low levels of reliable electricity (29%), adequate computing resources (29%), and stable internet connectivity (40%), alongside high frequencies of power outages (83%) and substantial energy cost burdens (75%), highlighting significant infrastructural constraints. In contrast, the high perceived benefit of solar-powered solutions (92%) suggests strong stakeholder support for renewable energy as a strategy to strengthen digital health implementation in low-resource settings.

Together, these findings illustrate the imbalance between existing infrastructural limitations and the considerable potential of renewable energy solutions to strengthen digital health implementation in low-resource settings.

#### Digital Infrastructure and Connectivity

Quantitative assessment of ICT infrastructure revealed substantial limitations in both hardware availability and internet connectivity. Only 29% of facilities reported adequate access to computers and related devices, while 71% indicated insufficient availability. Similarly, only 40% of facilities reported stable internet connectivity, while the majority (60%) experienced intermittent or unstable connections.

Qualitative data further illustrated how these limitations manifested in practice. Participants reported frequent disruptions to real-time data entry, delayed synchronization of health information systems, and constrained access to cloud-based platforms. In some facilities, limited device availability resulted in shared use among staff, contributing to workflow inefficiencies and delays in service delivery.

Notably, despite these constraints, participants highlighted the importance of adaptive system designs. Offline functionality was consistently identified as a critical enabler of continuity, allowing data capture during connectivity outages and subsequent synchronization upon restoration. This suggests that while infrastructural deficits remain significant, system-level design features can partially mitigate their impact.

#### Hardware Availability and System Performance

Hardware-related constraints emerged as a key limiting factor for digital health implementation. Quantitative findings showed that device shortages were widespread, while qualitative insights revealed additional challenges, including outdated or non-functional equipment and minimal access to maintenance services.

Although approximately 70% of facilities reported some level of technical support availability, this support was often limited in scope and reactive. Participants described delays in troubleshooting, a lack of spare parts, and insufficient preventive maintenance practices. These constraints collectively contributed to system downtime and reduced the reliability of digital health platforms.

The findings indicate that technical feasibility is determined not by individual components in isolation but by the interaction among multiple infrastructural elements. In particular, the interdependence of power supply, connectivity, and hardware availability emerged as a critical theme, with weaknesses in any one domain capable of undermining overall system performance.

### Economic Viability

#### Cost Burden and Financial Constraints

Quantitative analysis revealed that energy-related costs constitute a significant and unpredictable burden for health facilities. Approximately 75% of facilities reported difficulties managing electricity and fuel costs, particularly in contexts that require generator use. More than half of the facilities (58%) relied on diesel generators, leading to substantial recurring costs.

In addition, 67% of facilities reported incurring unplanned maintenance costs, often linked to power fluctuations and equipment damage. Fragmentation in digital health financing was also evident, with 50% of facilities indicating inconsistent funding for ICT infrastructure and system maintenance.

Qualitative findings reinforced these observations, with participants describing financial constraints as a major barrier to sustaining both energy and digital systems. High and unpredictable costs were reported to compete with other essential operational needs, resulting in deferred maintenance and underinvestment in infrastructure.

#### Perceived Economic Benefits of Solar Integration

Despite these financial challenges, both quantitative and qualitative data indicated strong perceived economic benefits associated with solar-powered solutions. Participants highlighted solar energy’s potential to reduce dependence on costly fuel-based systems, stabilize operational expenses, and improve long-term financial sustainability.

Facilities emphasized that although the initial capital investment in solar infrastructure may be substantial, the long-term cost savings and efficiency gains are significant. However, concerns remained about financing mechanisms, affordability, and the long-term sustainability of maintenance.

These findings suggest that while solar-powered digital health systems are economically promising, successful implementation will depend on viable financing models and long-term investment strategies.

### Health System Readiness

#### Workforce Capacity and Digital Readiness

Quantitative data indicate a moderately experienced workforce, with a concentration of staff in mid-level experience categories. However, qualitative findings revealed significant gaps in technical capacity, particularly in system maintenance, troubleshooting, and advanced digital skills.

Participants frequently reported the need for additional training and capacity-building initiatives to support the effective use and sustainability of digital health systems. Limited technical expertise at the facility level was identified as a constraint to both implementation and ongoing system optimization.

#### Organizational and Institutional Readiness

Findings suggest that while foundational elements for digital health adoption are present, overall institutional readiness remains uneven. Digital platforms, such as electronic medical record systems, provide a strong starting point; however, gaps in governance, financing, and infrastructure limit their effective use.

Facilities demonstrated varying levels of leadership support and engagement with digital health initiatives. While some institutions exhibited proactive engagement, others were constrained by limited strategic planning and resource allocation for digital health.

#### System Reliability and Operational Performance

Despite infrastructural and organizational challenges, digital health systems were generally perceived as beneficial and functional. Quantitative and qualitative findings indicate that system downtime, although present, was typically intermittent rather than constant. Participants reported improvements in patient record management and service efficiency associated with using digital systems.

However, these gains were described as fragile and highly dependent on the stability of the underlying infrastructure. Frequent disruptions to power and connectivity were identified as key factors limiting system reliability and performance.

### Integrated Mixed-Methods Findings

Integrating quantitative and qualitative results reveals a consistent, coherent pattern across facilities. Digital health systems are operational and valued, yet constrained by systemic infrastructural and financial limitations. Energy reliability, connectivity stability, and hardware availability are deeply interdependent and collectively shape system performance.

The findings demonstrate that the feasibility of solar-powered digital health solutions extends beyond technical deployment to encompass broader system-level considerations. While strong evidence supports readiness and acceptance, successful implementation will require coordinated investment across infrastructure, financing, and human capacity.

Overall, the results indicate that solar-powered digital health systems are feasible in low-resource settings, but their sustainability and scalability depend on addressing interconnected structural constraints within the health system. Table 3 presents qualitative themes and interpretations derived from thematic analysis. As the table summarizes qualitative findings rather than quantitative measures, counts and percentages were not applicable. The table has been reviewed and revised to improve clarity and interpretation of the thematic findings.

**Table 3.** Major Themes Identified Through Qualitative Analysis

Theme	Key findings	Interpretation
Power reliability and energy resilience	Frequent outages, reliance on generators, and limited solar coverage	Energy instability emerged as a major constraint on digital health system functionality and continuity
Digital infrastructure and connectivity	Unstable internet connectivity, delayed data synchronization, and limited real-time reporting	Connectivity limitations reduced system efficiency, data accessibility, and continuity
Hardware availability	Limited devices, outdated equipment, and inadequate maintenance support	Hardware constraints restricted the effective utilization of digital health systems
Economic constraints	High fuel costs, unpredictable electricity expenses, and limited operational budgets	Existing infrastructure arrangements may limit long-term financial sustainability
Perceived benefits of solar solutions	Improved reliability, reduced operating costs, and enhanced service continuity	Stakeholders expressed strong support for solar-powered digital health solutions
Workforce capacity and readiness	Moderate professional experience, but limited technical and maintenance skills	Capacity gaps may affect long-term sustainability and system maintenance
System performance and resilience	Systems remained functional but were vulnerable to infrastructure disruptions	Digital health systems were operational but lacked resilience to infrastructural challenges

## Discussion

### Principal Findings

This study provides a mixed-methods evaluation of the feasibility of solar-powered digital health systems in rural and peri-urban settings, demonstrating that while digital health platforms are increasingly present, their effectiveness is fundamentally constrained by infrastructural instability. These findings reinforce the argument that digital health implementation is not solely a technological issue but a complex systems challenge shaped by infrastructure, human capacity, and sociotechnical interactions.^1,4,8^

Quantitative findings reveal a critical mismatch between infrastructure access and functionality. Although most facilities are connected to the national electricity grid, reliability remains inadequate, resulting in frequent disruptions to digital health operations. This aligns with existing evidence showing that electricity reliability, rather than access alone, is a key determinant of health system performance in low-resource settings.^11^ The observed interruptions to electronic medical record systems and other services are consistent with prior studies demonstrating that unstable infrastructure compromises continuity of care and data integrity.^58-61^

Qualitative findings further contextualize these challenges, revealing that healthcare workers routinely rely on adaptive workarounds, such as reverting to paper-based systems or depending on generators. While these strategies enable short-term continuity, they introduce inefficiencies and potential safety risks, echoing previous research on unintended consequences of poorly supported health information technologies.^24,58^

Importantly, the integration of findings highlights that energy reliability represents the most critical bottleneck for digital health functionality, outweighing software-related limitations. This supports earlier work suggesting that digital health systems in low- and middle-income countries are fundamentally constrained by infrastructural readiness.^6,17,62^

### Comparison With Prior Work

The findings are consistent with global evidence emphasizing the foundational role of infrastructure in digital health adoption and sustainability. The World Health Organization has highlighted that digital health interventions require enabling environments, including reliable electricity, connectivity, and trained personnel.^2,4,5^ Similarly, studies on electronic medical record implementation have shown that infrastructural gaps significantly hinder effective use and scale-up.^6,17,62,63^

The high dependence on generators and associated financial burden observed in this study aligns with previous literature demonstrating the inefficiencies of fuel-based energy systems in healthcare settings.^11,14^ These systems not only increase operational costs but also introduce sustainability challenges, particularly in resource-constrained environments.^31,64^

The study also reinforces existing evidence on digital maturity gaps. Even where digital systems are introduced, their effectiveness is often limited by inadequate hardware, poor connectivity, and a lack of interoperability. Importantly, this study extends prior work by demonstrating that these factors are not independent but interdependent components of a broader system, consistent with sociotechnical perspectives.^8,65-68^

Furthermore, the strong stakeholder support for solar-powered solutions aligns with emerging research on renewable energy as a critical enabler of health system strengthening. Solar energy has been shown to improve service delivery, reduce costs, and enhance resilience in low-resource settings.^2,3^ These findings support the growing recognition that energy and digital health are intrinsically linked domains.

### Implications for Practice and Policy

The findings have several important implications for policy and practice.

First, digital health initiatives must be reframed as infrastructure-dependent interventions. Investments in software alone are insufficient without parallel investments in energy systems, connectivity, and hardware. This aligns with global recommendations advocating for integrated digital health strategies.^2,4,5,69^

Second, the results highlight the transformative potential of solar-powered infrastructure. By addressing energy instability, solar solutions can significantly enhance the reliability and performance of digital health systems, particularly in underserved settings.^3,14^

Third, the study underscores the need for sustainable financing mechanisms. High and unpredictable energy costs observed in this study reflect broader systemic challenges in health system financing. Innovative approaches, including public–private partnerships and blended financing models, will be essential to support implementation and scale-up.^31,64,70^

Fourth, capacity building emerges as a critical priority. Technical skill gaps and limited maintenance capacity were consistently identified as barriers to sustainability. Strengthening workforce capacity through training and support systems is essential for long-term success.^71,72^

Finally, the findings emphasize the importance of user-centered and context-sensitive design approaches. Digital health solutions must be tailored to local contexts, incorporating features such as offline functionality and modular design to enhance adaptability and resilience.^20,73-76^

### Theoretical and Conceptual Implications

This study contributes to theoretical discussions by reinforcing the relevance of sociotechnical systems theory in digital health implementation. The findings demonstrate that system performance is shaped by interactions between infrastructure, technology, and human factors, rather than by individual components in isolation.^65,77^

The results are also consistent with implementation science frameworks such as the NASSS framework, which emphasizes the complexity of adopting and scaling health technologies.^29,30^ Challenges identified in this study, particularly those related to infrastructure, financing, and organizational readiness, reflect multiple domains of complexity that influence implementation outcomes.

Additionally, the findings align with concepts of frugal and context-driven innovation, highlighting the importance of designing solutions that are adaptable to resource constraints.^75,76,78-81^ Solar-powered digital health systems represent a practical example of such innovation, integrating renewable energy with digital technologies to address systemic challenges.

### Strengths and Limitations

A key strength of this study is its mixed-methods design, which integrates quantitative and qualitative data to provide a comprehensive understanding of feasibility. This approach enhances the validity and depth of findings, consistent with best practices in health systems research.^34,35,54,55^

Including diverse facilities across multiple regions also improves the transferability of findings to similar low-resource settings. However, several limitations should be acknowledged. The sample size was relatively small, limiting generalizability. The quantitative analysis was descriptive, reflecting the exploratory nature of the study.^34,82,83^ Additionally, reliance on self-reported data may introduce bias. The study also did not include longitudinal evaluation or a detailed economic analysis, both of which would be necessary to assess long-term impact and cost-effectiveness. As with many mixed-methods studies, the relatively small quantitative sample limited statistical inference, while the qualitative component was intended to provide contextual depth and explanatory insights rather than population-level generalizability.

### Future Research

Future research should focus on longitudinal and implementation studies to evaluate the sustained impact of solar-powered digital health systems on service delivery and patient outcomes.^48,84^ There is also a need for robust economic evaluations to quantify cost-effectiveness and return on investment.^85,86^ Future research should also examine how sustainable energy infrastructure can support broader digital health and health system strengthening initiatives, including policy-driven approaches to improving healthcare delivery, service continuity, and population health outcomes.

Further research should examine the scalability, sustainability, and implementation of solar-powered digital health systems across diverse low-resource settings and health system contexts.^38,57^ Finally, studies examining governance, stakeholder engagement, and system integration will be essential to support sustainable implementation.^39,41,57^

## Conclusion

Solar-powered digital health systems represent a feasible and promising approach for strengthening healthcare delivery in low-resource settings. This study demonstrates that while digital health technologies are increasingly adopted, their effectiveness is often constrained by unreliable electricity, limited hardware availability, and inconsistent connectivity. The findings highlight energy reliability as a foundational requirement for sustainable digital health implementation and identify solar energy as a practical solution for improving system resilience and service continuity. However, successful implementation requires more than technological investment alone. Long-term sustainability depends on coordinated efforts to address financing, workforce capacity, maintenance systems, and organizational readiness. Integrating renewable energy solutions into broader digital health and health system strengthening strategies may help overcome persistent infrastructural barriers and support more reliable, scalable, and sustainable healthcare delivery in underserved settings.

## Supplemental Material

Supplemental material - Opportunities and Challenges of Implementing Solar-Powered Digital Health in Low-Resource Settings: A Mixed-Methods Study Supplemental material for Opportunities and Challenges of Implementing Solar-Powered Digital Health in Low-Resource Settings: A Mixed-Methods Study by Ronald Danny Nyatuka, Paul Macharia, and Md Shafiqur Rahman Jabin in The Journal of Health Care Organization, Provision, and Financing.

## Data Availability

The datasets generated and/or analyzed during the current study are not publicly available due to confidentiality agreements with participating facilities and ethical considerations, but are available from the corresponding author on reasonable request. All relevant aggregated data supporting the findings of this study are included within the article.*

## Appendix

### List of Abbreviations

EMR: Electronic Medical Record
FGD: Focus Group Discussion
ICT: Information and Communication Technology
LMICs: Low- and Middle-Income Countries
UHC: Universal Health Coverage
WHO: World Health Organization.
