# Supplementary material for: Opportunities and Challenges of Implementing Solar-Powered Digital Health in Low-Resource Settings: A Mixed-Methods Study
Source: Inquiry. 2026 Sep 1;63:00469580261485823. doi: 10.1177/00469580261485823 (PMC13535074; doi:10.1177/00469580261485823)
Supplement: Supplementary file 1 [file sj-pdf-1-inq-10.1177_00469580261485823.pdf]

S1 Table: Good Reporting of A Mixed Methods Study (GRAMMS) checklist

**Item**	**Reported**
Describe the justification for using a mixed methods approach to the research question	Study Design (pp. 4–5)
Describe the design in terms of the purpose, priority and sequence of methods	Study Design (pp. 4–5)
Describe each method in terms of sampling, data collection and analysis	Data Collection and Data Analysis sections (pp. 5–6)
Describe where integration has occurred, how it has occurred and who has participated in it	Integration of Mixed Methods and Integrated Mixed-Methods Analysis (p. 6)
Describe any limitation of one method associated with the present of the other method	Strengths and Limitations (p. 16)
Describe any insights gained from mixing or integrating methods	Integrated Mixed-Methods Findings and Discussion

Reference: O'Cathain A, Murphy E, Nicholl J. The quality of mixed methods studies in health services research. J Health Serv Res Policy. 2008;13(2):92-98.
